# Gamified assessment of cognitive performance during moderate hypoxia

**DOI:** 10.1371/journal.pone.0288201

**Published:** 2023-07-17

**Authors:** Jason M. Keeler, Erica Tourula, M. Jo Hite, Jennifer B. Listman, David J. Heeger, Nicholas L. Port, Zachary J. Schlader

**Affiliations:** 1 Department of Kinesiology, H.H. Morris Human Performance Laboratories, School of Public Health, Indiana University, Bloomington, Indiana, United States of America; 2 Statespace Labs, Inc. New York, New York, United States of America; 3 School of Optometry, Indiana University, Bloomington, Indiana, United States of America; University of Ljubljana, SLOVENIA

## Abstract

**Introduction:**

There is a need for rapid and objective assessment tools to identify people at risk of impaired cognitive function during hypoxia.

**Purpose:**

To test the hypotheses that performance on gamified cognitive tests examining the cognitive domains of executive function (Gridshot), working memory (Capacity) and spatial tracking (Multitracker) will be reduced during normobaric exposure to moderate normobaric hypoxia.

**Methods:**

Following three consecutive days of practice, twenty-one healthy adults (27 ± 5 y, 9 females) completed five 1-min rounds of the tablet-based games Gridshot, Capacity, and Multitracker (Statespace Labs, Inc.) at Baseline and 60 and 90 min after exposure to 14.0 ± 0.2% (hypoxia) and 20.6 ± 0.3% (normoxia) oxygen. Both conditions were completed on the same day and were administered in a single-blind, block randomized manner. Arterial oxyhemoglobin saturation was estimated via forehead pulse oximetry (SpO_2_). Data were analyzed using ANCOVA with a covariate of Baseline.

**Results:**

Compared to normoxia (98 ± 1%), SpO_2_ was lower (p < 0.001) at 60 (91 ± 3%) and 90 (91 ± 2%) min of hypoxia. No condition x time interaction effects were identified for any gamified cognitive tests (p ≥ 0.32). A main effect of condition was identified for Capacity (p = 0.05) and Multitracker (p = 0.04), but not Gridshot (p = 0.33). Post hoc analyses of the composite scores for both Capacity (p = 0.11) and Multitracker (p = 0.73) demonstrated no difference between conditions.

**Conclusion:**

Performance on gamified cognitive tests was not consistently affected by acute normobaric moderate hypoxic exposure.

## Introduction

Hypoxia, such as that experienced with high altitude exposure, often negatively impacts cognitive performance [1–3]. As the partial pressure of oxygen drops, so does the arterial blood oxygen partial pressure and the arterial blood oxygen-haemoglobin saturation. Decreases in arterial blood oxygen-haemoglobin saturation reflect reductions in oxygen delivery to bodily tissues, including the brain. Decreases in arterial blood oxygen-haemoglobin saturation cause a compensatory increase in cerebral blood flow, but this is not always sufficient to maintain optimal brain and cognitive function [4,5]. However due to confounding variables (i.e., exposure time, intensity of hypoxic stimulus, ambient temperature, barometric pressure, individual’s hypoxic symptoms) large variations in the effect of hypoxia on cognition have been reported [2,3,5]. For instance, the detrimental effects of acute hypoxia on simple cognitive tasks appear limited or unmeasurable until altitudes of 10,000–15,000 feet (3048–4572 m) or the sea level equivalent of the fraction of inspired oxygen (FiO_2_) of 14.3–11.8% [2,3]. However, complex or novel cognitive tasks maybe hindered at these altitudes/FiO_2_ levels or even with milder exposures [2,5]. These possible decrements are recognized by the use of supplemental oxygen for any U.S. Military flight in an unpressurized aircraft flying at altitudes between 10,000–12,000 feet (3048–3658 m) that last over an hour [6] and via hypoxia recognition trainings required for aviation aircrews [6,7]. This guidance aims to reduce the development of hypoxic symptoms of drowsiness, poor judgement, impaired coordination and efficiency, while also trying to train individuals to recognize their own hypoxic symptom signatures (set pattern of symptoms). Unfortunately, individuals often cannot recognize their own early symptoms of hypoxemia, despite prior exposure and training [2,8]. Therefore, the lack of early hypoxemia symptom recognition and the detrimental effects of hypoxia create a need to develop standardized assessments of cognitive function across multiple domains that can objectively identify crew members at risk of hypoxemia.

Investigations of cognitive function are evolving with the invention of new technologies and assessments [9]. The evolution of these technologies has led to the development of greater categorization of cognitive tasks by understanding new psycho-physiological pathways for cognitive processes and the development of cognitive mobile games [3,10]. Cognitive mobile games are designed to insert gameplay elements in non-gaming setting (i.e., cognitive assessments), to enhance engagement and intrinsic motivation with such assessments [11]. Gameplay elements can include elements like scoring, leaderboards, positive/negative feedback with sounds, and other elements seen in traditional videogame play. Indeed, cognitive mobile game assessments (i.e., gamified cognitive tests) may be an acute symptom screening tool for people who may encounter altered cognitive states in extreme environmental stressors, like hypoxia, due to the ease of play and ability to discern cognitive processes [12]. However, the use of gamified cognitive tests must provide assessments that are sensitive enough to detect early hypoxemia cognitive decrements, while also providing useful and rapid feedback to the end user.

Therefore, the primary purpose of this study was to evaluate whether three new gamified cognitive tests (Gridshot, Capacity, and Multitracker) designed as an offshoot to the video game training system Aim Lab© [13], are sensitive to identify alterations in cognitive function during exposure to moderate hypoxia. We tested the hypotheses that performance on gamified cognitive tests examining the cognitive domains of executive function (Gridshot), working memory (Capacity) and spatial tracking (Multitracker) will be reduced during normobaric exposure to an FiO_2_ = 14% compared to a normoxic exposure (FiO_2_ = 21%). Since physiological responses to a given FiO2 in moderate hypoxia often differs between people [2], a secondary purpose of the study was to understand how individual physiological responses (i.e., individualistic variability in arterial blood oxygen-haemoglobin saturation) to moderate hypoxia affect gamified cognitive test performance. Therefore, we tested the hypothesis that decreases in performance variables on gamified cognitive tests during moderate hypoxia, will be greater in individuals who experience a greater drop in arterial blood oxygen-haemoglobin saturation.

## Methods

### Experimental design

Before any study activities commenced, this study was approved by the Institutional Review Board at Indiana University (#12623) and was subsequently performed in accordance with the standards set by the latest revision of the Declaration of Helsinki, except for the registration in a database. This study utilized a single blind, block randomized, balanced, cross-over design. Participants reported to the laboratory on two separate occasions, with at least 72 hours between visits, and within 48 hours after the final practice day. Visit 1 included consenting, screening, and familiarization (practice gamified cognitive tests). Familiarization consisted of a demonstration of the gamified cognitive tests followed by 5 bouts of the the gamified cognitive tests. After each bout, the study personnel answered any questions. Following Visit 1, participants completed 1 practice session of the gamified cognitive tests for the three consecutive days immediately prior to returning to the lab for an experimental visit. A single practice session consisted of playing the gamified cognitive test bout 5 times. Familiarization and practice sessions were utilized to decrease the primary learning effects of the gamified cognitive tests, as previous work has demonstrated a learning plateau following 4 practice sessions [11,14]. Visit 2 was the experimental trial. Prior to participation all participants provided written informed consent after being fully informed of the experimental procedures and risks. Participants were recruited for this study from October 2021 until January 2022.

### Participants

An a priori power analysis was carried out with G-Power version 3.1.9.4 software. To our knowledge gamified cognitive tests have not been examined in hypoxia. Thus, we relied on previous work to estimate sample size for the current study, which revealed a Cohen’s d_z_ effect size of 0.78 with regards to the effect of moderate hypoxia on working memory performance [15]. Using this effect size and assuming a moderate positive correlation among repeated measures (r = 0.5), a sample size of at least 20 participants was needed to detect a significant interaction (condition x time) using standard parameters of 1-β = 0.80 and α = 0.05. Therefore, twenty-one healthy adults (27 ± 5y, 9 Females) were recruited and completed the study. Participant characteristics were height: 179 ± 10 cm, mass: 79 ± 21 kg, and body mass index: 25.5 ± 3.4 kg∙m^-2^. Participants self-reported being moderately active, nontobacco users, and free from any psychological, cardiovascular, and pulmonary diseases. Participants completed the Montreal Cognitive Assessment (MoCA) version 8.1 and had to score the age group median score of at least of 26 out of 30 to participate in the study (average score: 29 ± 2) [16]. Participants were also free of sickle cell trait and disease. Females self-reported to be normally menstruating and were confirmed not to be pregnant via pregnancy test. Females were tested across the menstrual cycle.

### Physiological instrumentation and measurements

Body mass (kg) and height (cm) were measured using a scale (Sauter, Balingen, Germany) and a stadiometer (Holtain Limited, Seritex, Wales, UK). Urine specific gravity was measured using a refractometer (Atago, Tokyo, Japan). Heart rate (bpm) and an estimate of arterial blood oxygen-haemoglobin saturation (S_p_O_2_, %) were measured continually via a pulse oximetry sensor (Nellcor N600x, Medtronic Inc., USA) placed on forehead. Blood pressure was measured using an automated blood pressure cuff (Suntech CT40, SunTech Medical, USA). Participants were also asked to identify the environment (normoxia or hypoxia) they were in at 60 and 90 min of exposure.

### Gamified cognitive tests

Participants completed gamified cognitive tests via a tablet (iPad, Apple Inc.) loaded with the Brain Lab application by Statespace Labs Inc., New York, NY, USA. Gamified cognitive tests were completed in bouts of 5 rounds of the three gamified cognitive tests (Gridshot, Capacity, and Multitracker). During experimental trials gamified cognitive tests were block randomized, so that there were 5 blocks with each block containing 1 round of each gamified cognitive test (Gridshot, Capacity, and Multitracker). Each gamified cognitive test is a 60-second test that provides immediate feedback to the participant upon completion by providing the player a composite score for the test. All gamified cognitive tests were completed in the upright, standing position, as orthostasis may provide a greater circulatory challenge [17]. During Gridshot, participants held either side of the tablet and tapped a button on the screen to shoot at targets, while utilizing the gyroscope technology to move through the visual field and aim the crosshairs at targets. During Capacity and Multitracker, participants placed the tablet on a stand and then tapped the screen with a finger to select correct targets. The stand was placed within arms length of the participant and at a comfortable height for the participants, which was maintained the same in all conditions with a participant.

Gridshot is a first-person shooter stylistic test that presents three targets of the same size simultaneously, with a new target appearing after one target is destroyed. The player destroys a target by aiming and shooting targets as quickly as possible. This test requires quick movements to align onto the target stimulus at small, medium, and long ranges. This test assesses the performance metrics of number of hits, misses, hit rate (percentage of shots attempts that successfully hit target, %), hits per second (number of targets successfully hit per second), shots per second, median time to hit (median time between consecutive hits, second), shot precision (the variability of the shot locations relative to the center of the targets) and a composite score (a weighted proprietary score based on the aforementioned metrics). Gridshot is further described by Listman et al. [11].

Capacity is a novel visuo-spatial working memory task that requires participants to complete a series of trials where multiple targets appear on the screen at once in varying colors. The targets disappear briefly and when they reappear, one target has changed color. The participants have to identify and tap the target that changed color. Difficulty of the test is modulated by the number of targets that appear on the screen through the addition or subtraction of one target based on a previous correct or incorrect response, respectively. This test assessed the performance metrics of number of correct trials completed, the number of incorrect trials completed, proportion of correct trials (the proportion of correct trials out of total trials completed, %), median time to hit (time from target reappearance to target selection, s), mean difficulty, and a composite score (a weighted proprietary score based on the aforementioned metrics). Capacity is described further by La Monica et al. [13].

Multitracker is a novel selective attention test that requires participants complete a series of trials, where multiple orbs (12) appear in the same color (Blue). A subset of the orbs (2–6) briefly change color (Red), marking them as the target orbs that the participant must track and identify at the end of the trial. After the orbs change back to blue, all orbs move randomly for a few seconds. When the orbs stop the participant is tasked with identifying the new location of each target orb by taping on each one individually. Difficulty of the test is modulated during the trials by changing orb movement speed (by increasing if participant identified all orbs correctly or decreasing speed if a distractor orb was selected) or by adding additional target orbs to be tracked. This test assessed the performance metrics of number of correct trials completed, the number of incorrect trials completed, proportion of correct trials (the proportion of correct trials out of total trials completed, %), median time to hit (time from reappearance to target selection, s), mean difficulty, and a composite score (a weighted proprietary score based on the aforementioned metrics).

Gameplay for all three gamified cognitive tests were the same during practice and experimental trials (as described above), except that an adaptive procedure was used to adjust task difficulty practice sessions to determine a personalized baseline level for Capacity and Multitracker. Specifically, the adaptive procedure manipulated variables included number of orbs for Capacity or movement speed of orbs for Multitracker. Difficulty was increased after each correct response and decreased after each incorrect response. During the experimental trials, in an effort to standardize performance, lab personnel reviewed the performances from practice session three and locked in a personalized baseline for all experimental trials.

### Visit 2: Experimental trial (see Fig 1)

**Fig 1:**
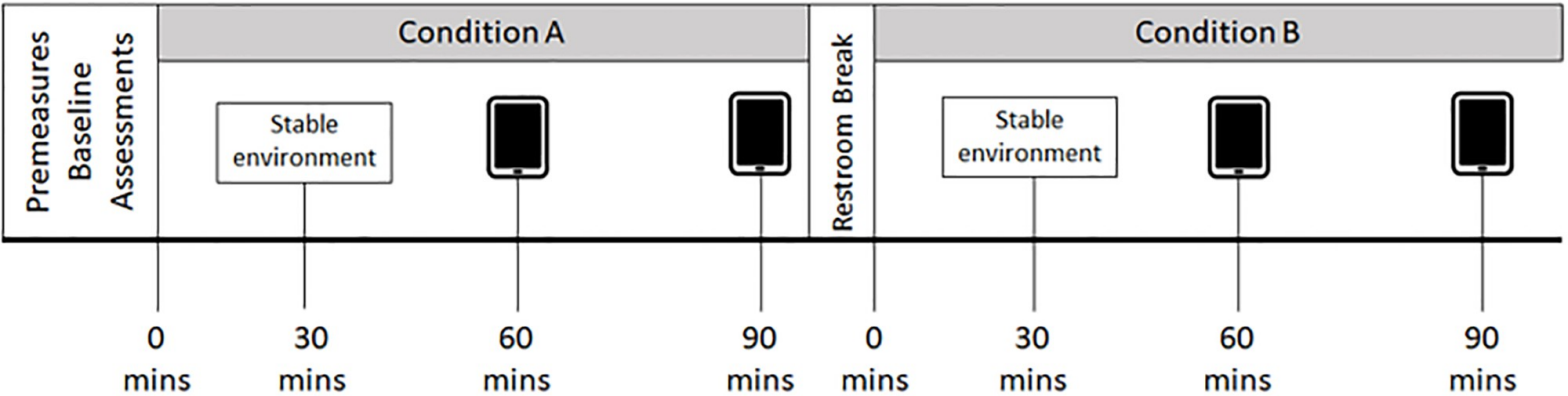
Visit 2: Experimental day timeline. Following baseline measures of body mass, heart rate, and arterial oxygen saturation, participants completed the baseline bout of gamified cognitive tests. Then participants sat in a chair in the environmental chamber and were allowed to watch a documentary between bouts of gamified cognitive tests. The environmental chamber was then set to a hypoxic (FiO_2_ = 14%) or normoxic (FiO_2_ = 21%) environment. During the hypoxic condition, participants would enter the environmental chamber at a FiO_2_ of 21%. Then the oxygen content in the room would be decreased over the first 30 min until it reached a stable FiO_2_ of 14%. Participants were blinded to order of exposure. The gamified cognitive test bouts were completed after 60 min of exposure to hypoxia or normoxia and again after 90 min of exposure. Following the 90 min assessment, participants were given a 5 min bathroom break and then reentered the chamber. The FiO_2_ in the calorimeter was changed to the opposite environmental condition. Again, participants completed the gamified cognitive test bouts after 60 min of exposure to the experimental conditions and again after 90 min of exposure. All participant data was upload to the cloud and participants were free to leave the lab.

Experimental trials were conducted on the same day, as it provided several benefits to study design via controlling for baseline stress, sleep, and hormonal changes due to normal menstruation, and lastly it provided convenience to the participants by decreasing participants time and travel burdens. Participants reported to a temperature-controlled laboratory (24.6 ± 1.4°C) for the experimental visit. A thermoneutral room temperature was utilized to decrease the effects of heat stress on cognition [3], which tends to be tied to increases of core body temperatures that occur at or above the limit of environmental exposure thermal equilibrium of 29.4°C [18]. Participants were instructed to refrain from strenuous exercise, consuming caffeine, and alcohol for 12 hours and food was restricted for 2 hours prior to the experimental visit. Upon arrival to the laboratory, a small sample of urine was collected from all participants to ensure participants were hydrated (i.e., urine specific gravity ≤1.020; measured urine specific gravity: 1.016 ± 0.01) and to confirm a negative pregnancy test for female participants. Laboratory personnel confirmed practice sessions were completed and uploaded to the server. Then baseline body mass and blood pressures were measured, and participants were instrumented for heart rate and S_p_O_2_. Subjects then completed 5 total bouts of gamified cognitive tests over the course of 4 hours.

### Visit 2 timeline (see Fig 1)

Following physiological baseline measurements, participants entered the 2.8 m^2^ environmental chamber to complete a baseline bout of the gamified cognitive tests in normoxia (FiO_2_ = 21%). After completing the bout of baseline gamified cognitive tests participants sat in a chair in the environmental chamber and were allowed to watch a documentary of their choosing between bouts of gamified cognitive tests. The environmental chamber was then set to a hypoxic (FiO_2_ = 14%) or normoxic (FiO_2_ = 21%) environment. A FiO_2_ = 14% was chosen as the literature is unclear of the effects of hypoxia at this level and there is also operational relevance as underpressurized aircraft can be operated at this hypoxic exposure [6], for extended periods of time. Participants were blinded to order of exposure and this was subsequently confirmed via questionnaire at 60 min and 90 min during each exposure. The gamified cognitive test bouts were completed after 60 min of exposure to hypoxia or normoxia and again after 90 min of exposure, to see if longer exposure impacts cognitive performance. Following the 90 min assessment, participants were given a 5 min bathroom break and then reentered the chamber. The FiO_2_ in the calorimeter was changed to the opposite environmental condition. Again, participants completed the gamified cognitive test bouts after 60 min of exposure to the experimental conditions and again after 90 min of exposure. During the hypoxic condition, participants would enter the environmental chamber at a FiO_2_ of 21%. Then the oxygen content in the room would be decreased over the first 30 min until it reached a stable FiO_2_ of 14%. All participant data were upload to a server and participants were free to leave the lab after deinstrumentation.

### Data and statistical analysis

Prior to analyses, all data were checked for outliers and the necessary assumptions for repeated measures ANOVA, Pearson’s Chi-square test, repeated measures ANCOVA, and linear mixed effects model analyeses and no corrections were necessary. Any learning effects were assessed via one-way repeated measures ANOVA of the composite scores from gamified cognitive test bouts during screening, practice day 1, practice day 2, practice day 3, and baseline on the experimental day. Post-hoc analysis was completed via Sidak’s multiple comparisons test. Test-retest reliability of each gamified cognitive test ws assessed via intraclass correlation analyses for practice day 3 trials. Comparisons of physiological variables (S_p_O_2_, HR) were assessed using two-way repeated measures ANOVA for condition (hypoxia and normoxia), time (60 and 90 min), and their interaction. If a significant main effect or interaction was observed, post-hoc tests using Sidak’s multiple comparison tests were completed. Pearson’s Chi-square test was used to assess subject blindedness to environmental conditions.

Consistent with testing our primary hypothesis that performance on gamified cognitive tests would be reduced during normobaric hypoxia compared to normoxic environmental conditions, a two-way repeated measures ANCOVA assessed the condition (hypoxia and normoxia), time (60 and 90 min), and their interaction effects on the available variables for each gamified cognitive test, with baseline values of gamified cognitive tests serving as the covariate. If a significant interaction was observed, pairwise comparions were made using post-hoc tests via Sidak’s multiple comparison tests. The primary outcome variables for this hypothesis were the composite scores for Gridshot, Capacity, and Multitracker, while the other performance metrics provided deeper understanding of the findings.

To assess our secondary hypothesis that decreases in performance metrics on gamified cognitive tests during moderate hypoxia will be greater in individuals who experience a greater drop in arterial oxygen saturation, a linear mixed effects model (lme4 and lmerTest R packages) with the variables of baseline values of gamified cognitive tests, individual decrease in S_p_O_2_ from baseline, time, and the interaction of the individual decrease in S_p_O_2_ from baseline and time. In these models, the time variable was transformed to log(time) because, based on large data sets from Aim Lab, player performance changes non-linearly over time within a day as well as across days and log(time) is the best approximation of the relationship between time and performance change (learning) when performance is not near the asymptote [15]. We utilized a type 3 sum of squares ANOVA table for significance of the individual decrease in S_p_O_2_ from baseline, time, and their interaction on the gamified cognitive test variables. The primary outcome variables for this hypothesis were the performance metrics from the gamified cognitive tests that were used to generate the composite scores for each game.

All data were analyzed using SPSS (IBM Corp. Released 2021. IBM SPSS Statistics for Windows, Version 28.0. Armonk, NY: IBM Corp), R version 4.1.3 (R foundation for Statistical Computing), and Prism software (Version 9.1.2, GraphPad Software Inc, La Jolla, CA, USA). A priori statistical significance was set at *P* ≤ 0.05, and actual p-values and partial eta squared (η_p_^2^) effect sizes are reported where possible. Group mean data are reported as mean ± standard deviation, and mean differences (diff.) and 95% confidence intervals (CI) are reported for multiple comparisons. Graphs show mean with individual values and 95% CI, unless otherwise stated.

## Results

### Practice sessions and learning effect

All participants completed 4 (screening, practice day 1, practice day 2, and practice day 3) gamified cognitive test game bouts prior to baseline experimental bout. Participants scores increased from screening to experimental baseline for all three gamified cognitive tests (p < 0.01). Gridshot composite scores increased (mean diff. 7311, 95% CI 4955, 9668, p < 0.01) from screening (22844 ± 3931) to experimental baseline (30156 ± 3662) trials. Gridshot composite scores increased with every successive day of practice (p < 0.05), with a final improvement seen from practice day 3 (27568 ± 3468) to experimental baseline (mean diff. 2588, 95% CI 1445, 3731, p < 0.01). Capacity composite scores increased (mean diff. 1755, 95% CI 618, 2893, p < 0.01) from screening (3367 ± 1289) to experimental baseline (5122 ± 2179) trials. Capacity composite score did not increase after practice day 2 (4646 ±2274), as experimental baseline and practice day 2 were not different (mean diff. 476, 95% CI -428, 1381, p = 0.70). Multitracker composite scores increased (mean diff. 6556, 95% CI 1692, 11421, p < 0.01) from screening (40861 ± 7486) to experimental baseline (47417 ± 6116) trials. Multitracker composite score at experimental baseline was not different from practice day 1 (44178 ± 6614, mean diff. 3239, 95% CI -1012, 7490, p = 0.24) or practice day 2 (44333 ± 3610, mean diff. 3084, 95% CI -575, 6742, p = 0.14). However, multitracker composite scores were increased at experimental baseline from practice day 3 (42737 ± 7763, mean diff. 4680, 95% CI 1193, 8167, p < 0.01). To investigate the test-retest reliability of each gamified cognitive test, an intraclass correlation analysis was done for the practice day 3 trials. Gridshot observed an average measure interclass correlation of 0.96. Capacity observed an average measure interclass correlation of 0.71. Multitracker observed an average measure interclass correlation of 0.78.

### Environmental and physiological variables

Participant S_p_O_2_ was greater at 60 mins of hypoxia compared to 90 mins of hypoxia (S_p_O_2(60 min)_: 91 ± 3%; S_p_O_2(90 min)_: 91 ± 2%, mean diff. 1%, 95% CI 1.1, 0.3, p < 0.01). However, no difference in S_p_O_2_ was seen between normoxia timepoints (S_p_O_2(60 min)_: 99 ± 1%; S_p_O_2(90 min)_: 99 ± 1%, mean diff. 0%, 95% CI -0.4, 0.4, p = 0.99). Participants had lower S_p_O_2_ during both hypoxia timepoints (S_p_O_2(60 min)_: 91 ± 3%; S_p_O_2(90 min)_: 91 ± 2%) compared to normoxia timepoints (S_p_O_2(60 min)_: 99 ± 1%, p < 0.01; S_p_O_2(90 min)_: 99 ± 1%, p < 0.01) with a mean difference between conditions of -8% (95% CI -9, -8) at 60 min and -7% (95% CI -8, -7) at 90 min. Heart rate was elevated during 90 min of hypoxia (88 ± 10 bpm) compared to 60 mins of hypoxia (84 ± 10 bpm, mean diff. 4, 95% CI 0, 7, p = 0.05). Heart rate did not differ between normoxia timepoints (HR_(60 min)_: 82 ± 11 bpm, HR_(90 min)_: 83 ± 9 bpm, mean diff. 2, 95% CI 2, 5, p < 0.01). Heart rate was not different between the conditions at 60 min (mean diff. 2, 95% CI -1, 6, p = 0.24), but was increased at 90 min during hypoxia compared to normoxia (mean diff. 4, 95% CI 1, 7, p = 0.02). Confirmation of blindness to environmental conditions was confirmed via Pearson’s Chi-square test (p = 0.89).

### Gamified cognitive tests: The effects of hypoxia

#### Gridshot (executive function test)

**Fig 2:**
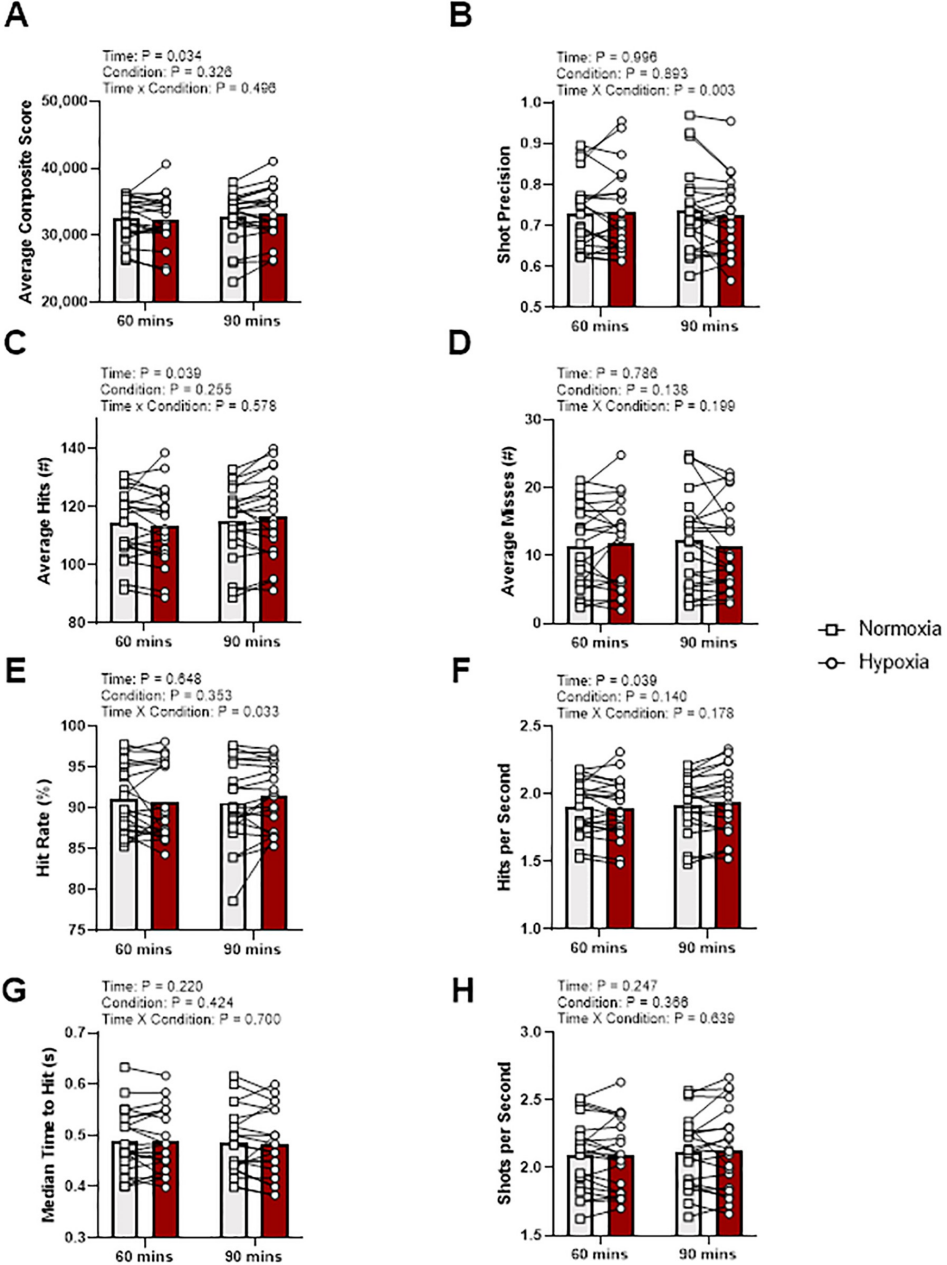
Gridshot output variables at 60 and 90 min during normoxia and hypoxia. Gray bars represents normoxia trials and red bars represent hypoxia trials. Data presented as means with individual responses (n = 21). P-values presented from F-tests for main effects and interaction from two-way repeated measures ANCOVA with baseline values serving as the covariate. (A) Composite score: Average composite score per round. (B) Shot precision: Average distance from target center. (C) Average hits: Average hits per round. (D) Average misses: Average misses per round. (E) Hit rate: Percentage of hits to total shots. (F) Hits per second: Average number of hits in one second. (G) Median time to hit: Average median time between two consecutive hits. (H) Shots per second: Average number of shots made per second.

For Gridshot (Fig 2), there were time by condition interaction effects for shot precision (p < 0.01, η_p_^2^ = 0.384) and hit rate (p = 0.03, η_p_^2^ = 0.217). However, there was not a time by condition interaction effect for composite score (p = 0.50, η_p_^2^ = 0.025), average hits (p = 0.58, η_p_^2^ = 0.017), average misses (p = 0.20, η_p_^2^ = 0.085), hits per second (p = 0.18, η_p_^2^ = 0.093), median time to hit (p = 0.70, η_p_^2^ = 0.008), and shots per second (p = 0.64, η_p_^2^ = 0.012). Pairwise comparisons of shot precision interaction showed no condition by time differences during normoxia (p = 0.85) or during hypoxia (p = 0.79). Pairwise comparisons of shot precision interaction showed no time by condition differences at 60 min (p > 0.73) or during 90 min (p > 0.90). Pairwise comparisons of hit rate interaction showed no condition by time differences during normoxia (p > 0.69) or during hypoxia (p ≥ 0.97). Pairwise comparisons of hit rate interaction showed no time by difference differences at 60 min (p > 0.72) or during 90 min (p > 0.51).

A main effect of time when controlling for baseline was identified for composite score (p = 0.03, η_p_^2^ = 0.216), average hits (p = 0.04, η_p_^2^ = 0.205) and hits per second (p = 0.04, η_p_^2^ = 0.205), however no other main effects of time, when controlling for baseline, were identified for shot precision (p = 0.99, η_p_^2^ = 0.000), average misses (p = 0.79, η_p_^2^ = 0.001), hit rate (%, p = 0.65, η_p_^2^ = 0.011), median time to hit (p = 0.22, η_p_^2^ = 0.078), or shots per second (p = 0.25, η_p_^2^ = 0.070). Pairwise comparison of composite score showed an increase (adj. mean diff. 535, 95% CI 63, 1007, p = 0.03) from 60 min (adj. mean 32431, 95% CI 31702, 33159) to 90 min (adj. mean 32966, 95% CI 32369, 33562), when controlling for baseline. Pairwise comparison of average hits showed an increase (adj. mean diff. 1.8, 95% CI 0.4, 3.2, p = 0.01) from 60 min (adj. mean 113.9, 95% CI 111.7, 116.2) to 90 min (adj. mean 115.7, 95% CI 113.6, 117.9), when controlling for baseline. Pairwise comparison of hits per second showed an increase (adj. mean diff. 0.03, 95% CI 0.01, 0.05, p = 0.01) from 60 min (adj. mean 1.90, 95% CI 1.86, 1.94) to 90 min (adj. mean 1.93, 95% CI 1.89, 1.97), when controlling for baseline.

There were no main effects of condition when controlling for baseline in composite score (p = 0.33, η_p_^2^ = 0.051), shot precision (p = 0.89, η_p_^2^ = 0.001), average hits (p = 0.26, η_p_^2^ = 0.068), average misses (p = 0.14, η_p_^2^ = 0.112), hit rate (p = 0.35, η_p_^2^ = 0.045), hits per second (p = 0.26, η_p_^2^ = 0.068), median time to hit (p = 0.42, η_p_^2^ = 0.034), and shots per second (p = 0.37, η_p_^2^ = 0.043).

#### Capacity (working memory test)

**Fig 3:**
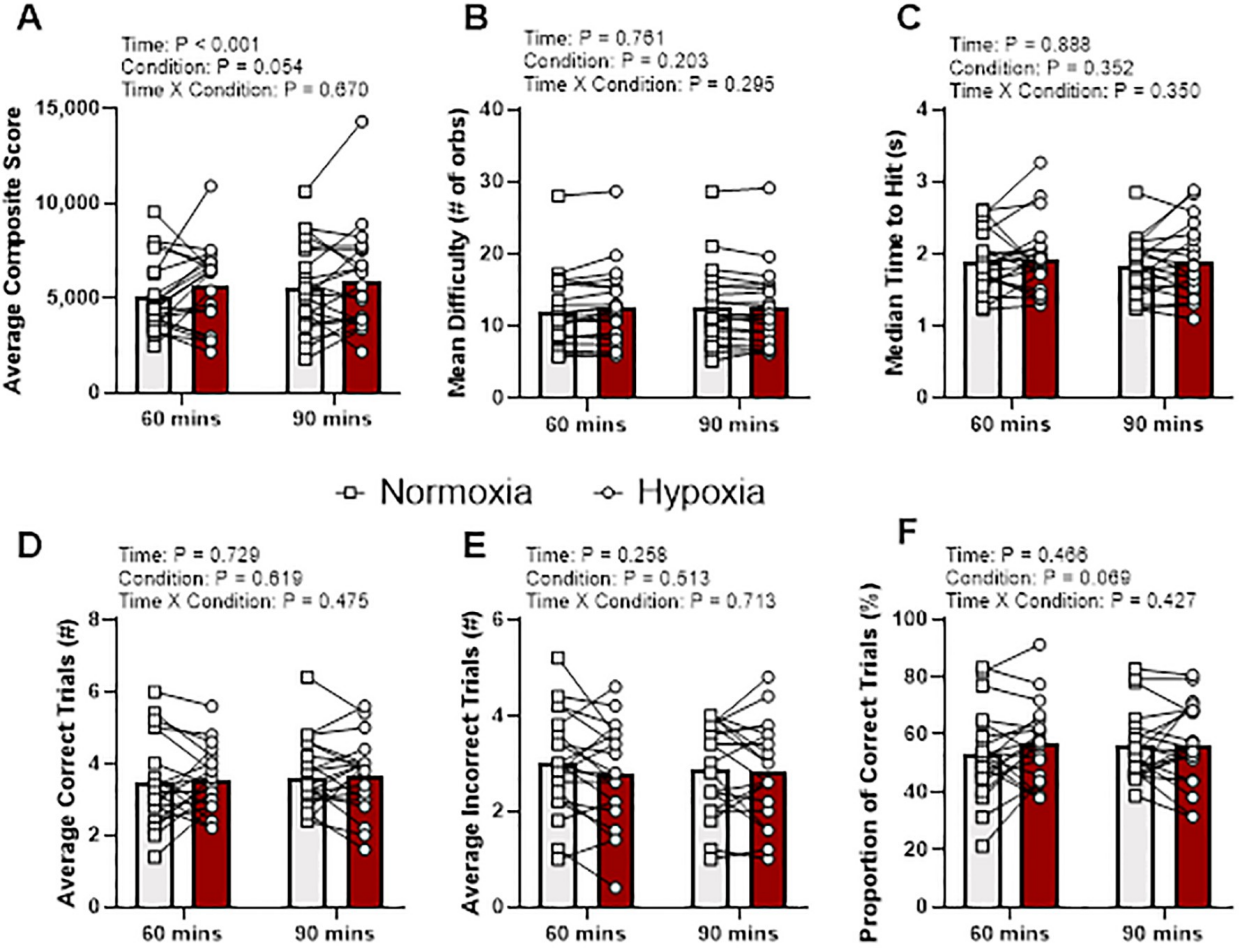
Capacity output variables at 60 and 90 min during normoxia and hypoxia. Gray bars represents normoxia trials and red bars represent hypoxia trials. Data presented as means with individual responses (n = 21). P-values presented from F-tests for main effects and interaction from two-way repeated measures ANCOVA with baseline values serving as the covariate. (A) Composite score: Average composite score per round. (B) Mean difficulty: Average difficulty during a round. (C) Median time to hit: Average median time between two consecutive hits. (D) Average Correct Trials: Average number of correct trials per round. (E) Average Incorrect Trials: Average number of incorrect trials per round. (F) Proportion of Correct Trials: Average percentage of correct trials within a round.

For Capacity (Fig 3), there were no time by condition interaction effects when controlling for baseline values for any variable: average composite score (p = 0.67, η_p_^2^ = 0.010), mean difficulty (p = 0.30, η_p_^2^ = 0.061), the median time to hit (p = 0.35, η_p_^2^ = 0.049), correct trials (p = 0.48, η_p_^2^ = 0.029), incorrect trials (p = 0.71, η_p_^2^ = 0.008), and proportion of correction trials (p = 0.43, η_p_^2^ = 0.035).

A main effect of time when controlling for baseline was identified for composite score (p < 0.01, η_p_^2^ = 0.47), however no other main effects of time, when controlling for baseline, were identified for mean difficulty (p = 0.76, η_p_^2^ = 0.005), the median time to hit (p = 0.89, η_p_^2^ = 0.001), correct trials (p = 0.73, η_p_^2^ = 0.007), incorrect trials (p = 0.26, η_p_^2^ = 0.070), and proportion of correction trials (p = 0.47, η_p_^2^ = 0.030). Composite score increased (adj. mean diff. 419, 95% CI 30, 808, p = 0.04) from time 60 min (adj. mean 5327, 95% CI 4790, 5864) to 90 min (adj. mean 5747, 95% CI 5257, 6236), when controlling for baseline.

A main effect of condition when controlling for baseline was identified for composite score (p = 0.05, η_p_^2^ = 0.061), while no other main effects of condition when controlling for baselines were identified for mean difficulty (p = 0.30, η_p_^2^ = 0.061), median time to hit (p = 0.30, η_p_^2^ = 0.061), correct trials (p = 0.60, η_p_^2^ = 0.014), incorrect trials (p = 0.51, η_p_^2^ = 0.024), or proportion of correct trials (p = 0.30, η_p_^2^ = 0.061). Pairwise comparisons of composite score showed no change (adj. mean diff. 454, 95% CI -112, 1020, p = 0.11) from normoxia (adj. mean 5310, 95% CI 4697, 5923) to hypoxia (adj. mean 5764, 95% CI 5277, 6250), when controlling for baseline.

#### Multitracker (spatial tracking)

**Fig 4:**
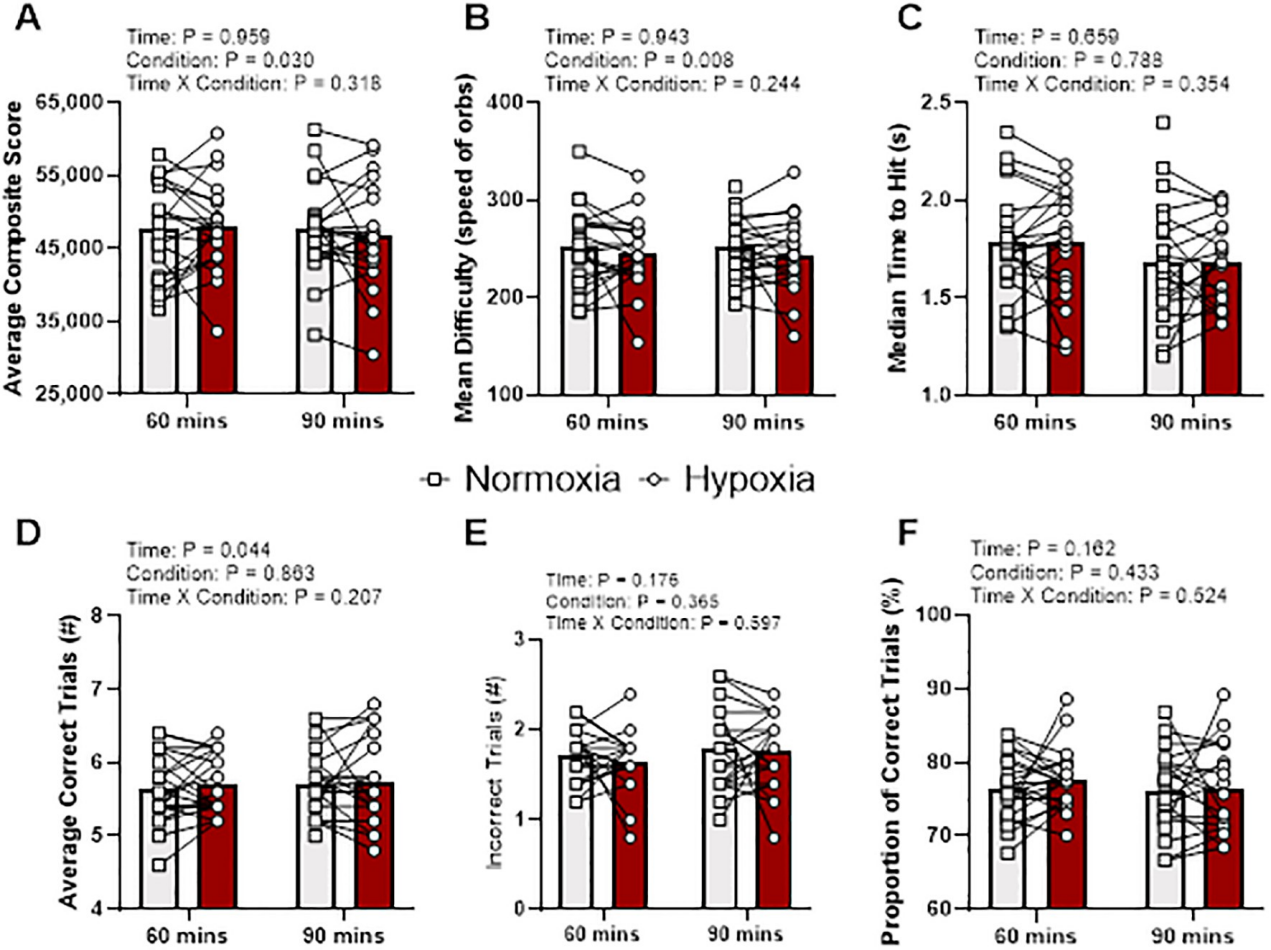
Multitracker output variables at 60 and 90 min during normoxia and hypoxia. Gray bars represents normoxia trials and red bars represent hypoxia trials. Data presented as means with individual responses (n = 21). P-values presented from F-tests for main effects and interaction from two-way repeated measures ANCOVA with baseline values serving as the covariate. (A) Composite score: Average composite score per round. (B) Mean difficulty: Average difficulty during a round. (C) Median time to hit: Average median time between two consecutive hits. (D) Average Correct Trials: Average number of correct trials per round. (E) Average Incorrect Trials: Average number of incorrect trials per round. (F) Proportion of Correct Trials: Average percentage of correct trials within a round.

For Multitracker (Fig 4), there were no time by condition interaction effects for any variable: average composite score (p = 0.32, η_p_^2^ = 0.052), mean difficulty (p = 0.24, η_p_^2^ = 0.075), the median time to hit (p = 0.35, η_p_^2^ = 0.048), correct trials (p = 0.21, η_p_^2^ = 0.087), incorrect trials (p = 0.60, η_p_^2^ = 0.022), and proportion of correction trials (p = 0.52, η_p_^2^ = 0.023).

A main effect of time was identified for correct trial (p = 0.04, η_p_^2^ = 0.206), however no other main effects of time were identified for average composite score (p = 0.96, η_p_^2^ < 0.001), mean difficulty (p = 0.94, η_p_^2^ < 0.001), the median time to hit (p = 0.66, η_p_^2^ = 0.011), incorrect trials (p = 0.18, η_p_^2^ = 0.099), and proportion of correction trials (p = 0.16, η_p_^2^ = 0.105). Pairwise comparison of correct trials showed no change (adj. mean diff. -0.05, 95% CI -0.16, 0.06, p = 0.36) from 60 min (adj. mean 5.69, 95% CI 5.54, 5.84) to 90 min (adj. mean 5.74, 95% CI 5.60, 5.88), when controlling for baseline.

Main effects of condition were identified for composite score (p = 0.03, η_p_^2^ = 0.225) and mean difficulty (p < 0.01, η_p_^2^ = 0.331), while no other condition effects identified for median time to hit (p = 0.79, η_p_^2^ = 0.004), correct trials (p = 0.86, η_p_^2^ = 0.002), incorrect trials (p = 0.37, η_p_^2^ = 0.046), and proportion of correction trials (p = 0.43, η_p_^2^ = 0.035). Pairwise comparison of composite score showed no change (adj. mean diff. -325, 95% CI -2255, 1604, p = 0.73) from normoxia (adj. mean 47667, 95% CI 45151, 50183) to hypoxia (adj. mean 47342, 95% CI 45695, 48988), when controlling for baseline. Pairwise comparison of mean difficulty showed no change (adj. mean diff. -6, 95% CI -17, 5, p = 0.29) from normoxia (adj. mean 249, 95% CI 235, 264) to hypoxia (adj. mean 244, 95% CI 233, 254), when controlling for baseline.

### Gamified cognitive tests: The effects of individiaul S_p_O_2_ decrements on performance matrics

The interaction term was not significant for all gridshot performance metrics (Table 1). Capacity performance metrics showed no significant effects of S_p_O_2_ decrease or its interaction with Time (Table 2). The Capacity performance metric of Correct Trials demonstrated a time effect, such that participants improved at subsequent time points irrespective of decreases in S_p_O_2_. Multitracker performance metrics showed no significant effects of S_p_O_2_ decrease or its interaction with Time (Table 3). The Multitracker performance metrics of Median Time to Hit and Mean Difficulty demonstrated time effects, such that participants improved at subsequent time points irrespective of decreases in S_p_O_2_.

**Table 1:** Gridshot linear mixed effects model estimate tables for each performance metric.

Predictors	Hits			Misses			Hit Rate			Hits per Second			Shots Per Second			Median time to hit			Shot Precision		
	Estimates	CI	p	Estimates	CI	p	Estimates	CI	p	Estimates	CI	p	Estimates	CI	p	Estimates	CI	p	Estimates	CI	p
(Intercept)	32.81	-63.46–129.08	0.499	0.16	-0.15–0.47	0.305	0.08	-0.31–0.47	0.676	0.06	-0.02–0.14	0.116	8.83	-10.17–27.83	0.357	0.30	0.12–0.48	0.001	0.16	0.04–0.27	0.009
Baseline	1.00	0.83–1.18	< 0.001	0.97	0.80–1.14	< 0.001	1.01	0.82–1.20	<0.001	0.83	0.68–0.97	<0.001	0.78	0.54–1.02	< 0.001	0.68	0.49–0.87	< 0.001	0.76	0.61–0.92	<0.001
Time	2.24	-4.81–9.28	0.529	0.01	-0.01–0.03	0.302	0.01	-0.01–0.03	0.241	-0.00	-0.01–0.00	0.093	1.31	-2.44–5.05	0.489	-0.00	-0.01–0.00	0.574	0.01	-0.00–0.02	0.178
S_a_O_2_ Decrease	-0.71	-4.58–3.17	0.717	-0.00	-0.01–0.01	0.458	-0.00	-0.02–0.01	0.533	-0.00	-0.00–0.00	0.627	0.34	-1.77–2.44	0.750	-0.00	-0.00–0.00	0.676	0.00	-0.00–0.01	0.238
Time*S_a_O_2_ Decrease	0.31	-1.09–1.71	0.662	0.00	-0.00–0.01	0.292	0.00	-0.00–0.01	0.344	0.00	-0.00–0.00	0.914	-0.10	-0.86–0.67	0.803	0.00	-0.00–0.00	0.735	-0.00	-0.00–0.00	0.122
Random Effects																					
σ^2^	483.08			0.00			0.00			0.00			120.94			0.00			0.00		
Τ_00_	463.90			0.01			0.01			0.00			300.96			0.00			0.00		
ICC	0.49			0.63			0.72			0.65			0.71			0.63			0.50		
*N*	21			21			21			21			21			21			21		
Observations	84			84			84			84			84			84			84		
Marginal R^2^ / conditional R^2^	0.792 / 0.894			0.819 / 0.933			0.805 / 0.946			0.822 / 0.937			0.608 / 0.888			0.627 / 0.861			0.753 / 0.877		

Model structures for each performance metric with main and interaction effect coefficient estimates (Estimates) as a measure of their individual effect size on the outcome measures, confidence intervals (CI), and a p-value. Significance was set at p < 0.05.

**Table 2:** Capacity linear mixed effects model estimate tables for each performance metric.

Predictors	Number of Correct Trials			Number of Incorrect Trials			Proportion of Correct Trials			Median Time to Hit			Mean difficulty		
	Estimates	CI	P	Estimates	CI	P	Estimates	CI	P	Estimates	CI	P	Estimates	CI	p
(Intercept)	7.02	1.91–12.13	0.008	0.75	0.11–1.38	0.022	0.64	-2.49–3.76	0.687	4.40	-0.60–9.39	0.084	0.24	0.06–0.42	0.008
Baseline	0.52	0.24–0.80	<0.001	0.66	0.37–0.96	<0.001	0.94	0.71–1.18	<0.001	0.74	0.45–1.04	<0.001	0.50	0.18–0.81	0.002
Time	0.85	0.00–1.70	0.049	-0.05	-0.13–0.02	0.156	0.14	-0.09–0.36	0.240	-0.24	-1.03–0.54	0.542	0.02	-0.01–0.04	0.180
SpO2 Decrease	-0.04	-0.52–0.44	0.877	-0.00	-0.04–0.04	0.921	0.02	-0.11–0.15	0.789	-0.04	-0.48–0.40	0.871	0.00	-0.01–0.02	0.693
Time*SpO2 Decrease	0.03	-0.14–0.21	0.730	0.00	-0.01–0.02	0.947	-0.00	-0.05–0.04	0.851	-0.02	-0.18–0.14	0.834	0.00	-0.01–0.01	0.994
Random Effects															
σ^2^	6.35			0.05			0.41			5.62			0.01		
Τ_00_	11.70			0.07			6.63			8.48			0.01		
ICC	0.65			0.60			0.94			0.60			0.62		
*N*	21			21			21			21			21		
Observations	83			83			83			83			83		
Marginal R^2^ / conditional R^2^	0.358 / 0.774			0.424 / 0.767			0.751 / 0.986			0.465 / 0.787			0.276 / 0.723		

Model structures for each performance metric with main and interaction effect coefficient estimates (Estimates) as a measure of their individual effect size on the outcome measures, confidence intervals (CI), and a p-value. Significance was set at p < 0.05.

**Table 3:** Multitracker linear mixed effects model estimate tables for each performance metric.

Predictors	Number of Correct Trials			Number of Incorrect Trials			Proportion of Correct Trials			Median Time to Hit			Mean Difficulty		
	Estimates	CI	p	Estimates	CI	p	Estimates	CI	p	Estimates	CI	p	Estimates	CI	p
(Intercept)	13.43	6.43–20.43	**<0.001**	0.62	0.19–1.04	**0.005**	81.82	13.30–150.34	**0.020**	4.14	1.29–6.99	**0.005**	0.39	0.20–0.59	**<0.001**
Baseline	0.53	0.27–0.79	**<0.001**	0.68	0.48–0.89	**<0.001**	0.60	0.35–0.86	**<0.001**	0.49	0.18–0.80	**0.002**	0.49	0.24–0.75	**<0.001**
Time	0.16	-0.34–0.65	0.528	-0.07	-0.11 –-0.03	**<0.001**	7.15	0.36–13.94	**0.039**	0.29	-0.14–0.72	0.183	-0.00	-0.02–0.01	0.391
SpO2 Decrease	0.02	-0.26–0.29	0.895	-0.00	-0.02–0.02	0.777	1.63	-2.11–5.37	0.388	-0.06	-0.30–0.17	0.578	0.00	-0.00–0.01	0.580
Time*SpO2 Decrease	-0.00	-0.10–0.10	0.995	0.00	-0.01–0.01	0.875	-0.80	-2.16–0.56	0.245	0.02	-0.06–0.11	0.597	-0.00	-0.00–0.00	0.627
Random Effects															
σ^2^	2.28			0.01			454.74			2.00			0.00		
Τ_00_	1.78			0.01			377.34			0.80			0.00		
ICC	0.44			0.51			0.45			0.28			0.25		
*N*	21			21			21			21			21		
Observations	83			83			83			83			83		
Marginal R^2^ / conditional R^2^	0.339 / 0.629			0.613 / 0.809			0.383 / 0.663			0.242 / 0.458			0.286 / 0.461		

Model structures for each performance metric with main and interaction effect coefficient estimates (Estimates) as a measure of their individual effect size on the outcome measures, confidence intervals (CI), and a p-value. Significance was set at p < 0.05.

## Discussion

In opposition to our hypothesis the composite scores of the three gamified cognitive tests did not detect changes in cognition during moderate hypoxia. Interestingly, Mulitracker (spatial tracking) demonstrated a main effect of condition and agreed with our hypothesis of decrements in performance with moderate hypoxia exposure, while Capacity (working memory) showed a main effect of condition with slight improvements in composite scores during hypoxia exposure. Unfortunately, post hoc analyses of the composite scores for both Multitracker and Capacity demonstrated no difference between conditions. This, along with the lack of detectable cognitive change in Gridshot (executive function), continues to demonstrate how difficult it is to measure the cognitive effects of moderate hypoxia, as noted by others [1,3,15,19]. Indeed, measuring the cognitive effects of moderate hypoxia is difficult, but the current work provides sufficient evidence to warrant further investigation to gamified cognitive tests to detect changes, as main effects of condition were seen in composite scores and individual component variables that were used to derive the composite scores (Figs 2–4).

The premise of this study was to determine if the gamified cognitive tests could provide rapid and objective measures to detect cognitive impairments for personnel during exposure to moderate altitude. While the gamified cognitive tests did provide rapid feedback via a composite score following a 60 second test, there was no clear delineation between normoxic and hypoxic trials. The gamified cognitive tests were designed to asses complex tasks, which are at greater risk of impairment compared to simple tasks at lower hypoxic stress levels [3,15,20,21]. Unfortunately, this study aligns with current knowledge for acute moderate hypoxic (F_i_O_2_ between 14.3–11.8%) exposure’s effects on complex cognitive tasks, which has been mixed when using a variety of validated cognitive tests. These mixed results are best outlined in reviews by Taylor et al. [3] and Virués-Ortega et al. [5] where various exposure times, hypoxic stress levels, and cognitive tests were applied with varied outcomes of either slight improvements, no change, or decrements in cognitive function.

The lack of congruency among previous investigations and the current study may demonstrate the individuality of hypoxic symptom signatures during acute moderate hypoxic stress. Specifically, looking at the individual data points (Figs 2–4) there are no clear set of patterns observed in any of the composite scores for the three gamified cognitive tests. Additionally, to investigate the individualized responses to hypoxia, the decrease from baseline S_p_O_2_ was utitlized as a potential marker to explain an individual’s response to hypoxia. That said, the current study did not demonstrate any effect of changes in S_p_O_2_ on the measured performance metrics (Tables 1–3), although shot precision (p = 0.122) and misses (p = 0.292) in Gridshot (Table 1) likely warrant further investigation. Nevertheless, the lack of apparent effects provides evidence of the individualistic responses to hypoxemia, which are brought about via exposure to hypoxia and depend on a myriad of factors including but not limited to genetic predisposition, prior exposures, acclimatization, daily physiological conditions (e.g., hydration, fatigue, etc.), and other factors [22]. Previous research in pilots demonstrates that the individual may not recognize their own symptoms during exposure [8] but creating an objective tool such as a personalized gamified cognitive test could provide immediate feedback to personnel working at altitude. Therefore, further research into hypoxic symptom signatures could yield valuable information on the determination of an individual’s response to hypoxemia, which then could be used to develop a targeted rapid and object assessment. Specifically, gamified cognitive tests tailored to the known domains of impairment first observed in an individual’s hypoxic signature could be assigned as the specific test battery for the individual. This would create a more precise and personalized approach to evaluating cognitive decrements during hypoxic exposure and provide enhanced safety to personnel who work in such environments. This personalized approach to evaluating early signs of moderate hypoxic cognitive impairment with gamified cognitive tests warrants further investigation.

### Experimental considerations

There are a few experimental considerations to consider for this investigation. First, our participants were exposed to normobaric hypoxia, which can physiologically differ from hypobaric hypoxia [2]. Specifically, one study found that at spending 40 min at 4,500 m in hypobaric hypoxia elicited greater hypoxemia, hypocapnia, blood alkalosis and heart rate, compared to normobaric hypoxia of an equivalent partial pressure of O_2_ [23]. That said, while differences between normobaric and hypobaric hypoxia are measureable, there tends to be slight differences and therefore we believe a normobaric hypoxia was acceptable for this experiment. A second consideration for this study is the use of tablet based gamified cognitive tests. This is a new method of evaluation, which was applied to provide a tangible and readily available tool for measurements. Thirdly, the gamified cognitive tests were designed for the sophisticated analysis of a multitude of parameters to evaluate cognitive performance, which could increase the risk of type 1 error. Therefore, a priori statistical designs and conservative conclusions were utilized for each hypothesis. Lastly, the experimental design did not incorporate physical work (or exercise) and therefore would be more applicable to pilots or personnel working in relatively stationary locations. The addition of muscular work would further decrease oxygen saturation and presumably impair cognitive function to a greater extent. This study was designed as a proof of concept, and further research would be needed prior to implementation in a working environment.

### Perspectives

Workers or military personel exposed to low oxygen environments, such as altitude, are at risk of cognitive impairments. However, a variety of factors (fraction of inspired O_2_, time of exposure, acclimation status, etc.) determine the degree to which cognitive function is affected [2]. While severe hypoxia is characterized by easily recognizable signs and symptoms such as headache, nausea, and fatigue, the signs and symptoms of moderate hypoxia are harder to identify. This lack of clear identifiable characteristics has been demonstrated in multiple studies that have an array of signs and symptoms, which may be due to early individualized hypoxic symptom signatures. However, as many jobs necessitate exposure to moderate altitude a valid, rapid, and objective measure of cognitive function is needed to identify personnel at risk of impaired job performance and deleterious health outcomes. This is highlighted in a study of pilots and aircrews who were constantly exposed to moderate hypoxia and have demonstrated impairments in simulated flight performance [24]. Unfortunately, the current study was unable to reliably ascertain measurable decrements in cognitive function via the current gamified cognitive tests. However, two out of the three games demonstrated slight cognitive changes between normoxia and hypoxia, based on main effect comparisons. Thus, further research is required to identify the utility of gamified cognitive tests to detect changes in cognitive function during moderate hypoxia.

## Conclusion

The gamified cognitive tests of Gridshot (executive function), Capacity (working memory), and Multitracker (spatial tracking) did not consistently detect alterations in cognitive function during exposure to 90 min of moderate hypoxia. However, further research of gamified cognitive tests that integrate cross domain functions may yield fruitful and rapid tests of impaired cognitive function due to environmental stressors.
